# GC-MS Analysis, Antioxidant and Antifungal Studies of Different Extracts of *Chaetomium globosum* Isolated from *Urginea indica*

**DOI:** 10.1155/2022/1388850

**Published:** 2022-12-24

**Authors:** Shailja Kumari, Swati Kumari, Chandrika Attri, Ruchi Sharma, Sourabh Kulshreshtha, Taoufiq Benali, Abdelhakim Bouyahya, Eda Sönmez Gürer, Javad Sharifi-Rad

**Affiliations:** ^1^Faculty of Applied Sciences and Biotechnology, Shoolini University of Biotechnology and Management Sciences, Solan, Himachal Pradesh, India; ^2^School of Bioengineering & Food Technology, Shoolini University of Biotechnology and Management Sciences, Solan, 173229 Himachal Pradesh, India; ^3^Environment and Health Team, Polydisciplinary Faculty of Safi, Cadi Ayyad University, Sidi Bouzid B.P. 4162, Morocco; ^4^Laboratory of Human Pathologies Biology, Department of Biology, Faculty of Sciences, And Genomic Center of Human Pathologies, Mohammed V University, Rabat 10106, Morocco; ^5^Sivas Cumhuriyet University, Faculty of Pharmacy, Department of Pharmacognosy, Sivas, Turkey; ^6^Facultad de Medicina, Universidad del Azuay, Cuenca, Ecuador

## Abstract

To discover new natural resources with biological effects, the chemical investigation of antioxidant and antimicrobial activities of extract's *Chaetomium globosum* isolated from roots of *Urginea indica*. Gas chromatography-mass spectrometry (GC-MS) analysis demonstrated the presence of the major chemical constituents present in the methanol extract (1,3-oxathiolane, 1,3-cyclopentadiene, 5-(1-methylethylidene), 5,9-hexadecadienoic acid, methyl ester, decane), chloroform extract (acetic acid, diethoxy-, ethyl ester, 2,2-bis(ethylsulfonyl)propane, 3-methyl-2-(2-oxopropyl) furan), and hexane extract (3-hexanone, 4,4-dimethyl, decane,2,6-dimethyldecane, decane, 2,4,6-trimethyl, decane, 2,4,6-trimethyl, 1-butanesulfinamide, 1,1,2,2,3,3,4,4,4-nonafluoro-N-methyl, decane). The total compound identified (56.2%) in chloroform extract, (54.72%) in hexane extract, and (65%) in methanol extract. The antioxidant effects were performed using diphenylpicrylhydrazyl radical (DPPH). The results showed that the methanol extract showed significantly the highest anti-DPPH with an IC_50_ value of 37.61 ± 1.37 *μ*g/mL, followed by chloroform and hexane extracts with IC_50_ values of 40.82 ± 3.60 and 45.20 ± 2.54 *μ*g/mL, respectively. The antifungal activity of extracts was evaluated against pathogens fungi including *Fusarium oxysporum*, *Rosellinia necatrix*, *Cladosporium xanthochromaticum*, and *Sclerotinia sclerotiorum*. Methanolic and chloroform extracts showed maximum inhibition against all test pathogens, while hexane extract showed minimum inhibition.

## 1. Introduction

Endophytes are microorganisms, frequently bacteria or fungi, that live within a plant for at least part of its life cycle without causing discernable disease. Usually, endophytic fungi are extensively dispersed in all kingdoms of the plant kingdoms. They generate advantageous plant secondary metabolites and utilize them for medical, agricultural, and industrial usage. Endophytic fungi are a new type of microbes isolated from plants that have received attention due to their ecological nature [1]. Endophytes are the repository of new and unique secondary metabolites; these metabolites can serve as a marvelous origin of drugs for anti-inflammatory, antioxidant, anticancer, antimicrobial, and antidiabetic activities. In current years, mushrooms known as higher fungi are a rich source of secondary metabolites as compared to metabolites derived from plants. There are three categories of mushrooms, saprotrophic, parasitic, and symbiotic. There are millions of species of mushrooms but only 4% of species are considered to be safe for edible. Nowadays, natural products from mushrooms are gaining the attention of researchers. Secondary metabolites of higher fungi (mushrooms) are an underexplored resource compared to plant-derived secondary metabolites. An increasing interest in mushroom natural products has been noted in recent years. Mushrooms are more nutritious, but other than nutrition mushrooms are also proficient in producing valuable bioactive secondary metabolites, these metabolites have great uses in the medicinal field. There are different categories of bioactive metabolites produced from mushrooms such as lectins, polysaccharides, phenolic, terpenoids, polyphenolics, and other organic compounds like ceramides. These metabolites possess immunomodulating properties. Anticancer compound 9-methoxycamptothecin is isolated from endophytic fungi. Taxol, which is a potent anticancer compound, widely used for the treatment of different types of cancer, produces by different species of endophytic fungi isolated from different medicinal plants. Vinblastine is an anticancer compound produced by the endophytic fungi *Alternaria* sp. These are the major anticancer metabolites produced by endophytes. All over the world, cancer is the main killer disease. Every year, more than six billion people suffer from cancer, and the discovery of these fungal endophytes is a great achievement for human welfare. These metabolites have remarkable biological and industrial applications. Secondary metabolites produced *via* endophytic fungi bear in various fascinating activities useful in different fields.

*C. globosum* endophytic strain isolated from *Ginkgo biloba* inhibited the growth of pathogens and acted as a strong biocontrol agent [2]. In another study, nine new cytochalasan alkaloids were isolated from the *C. globosum* strain obtained from a common pillbug (*Armadillidium vulgare*). These cytochalasins showed the capability to inhibit cancer cells [3]. Chaetoviridin A, a strong antifungal compound isolated from *Chaetomium globosum*, inhibits the growth of *Verticillium dahlia* (a soil-born pathogen) causing disease in the cotton plant. The inhibition of this pathogen improves the yield of the cotton plant [4]. Medicinal plants generally possess endophytes with comparable secondary metabolites and therapeutic activities. *Urginea indica* is a distinguished Indian medicinal plant with various medicinal properties. With this motivation, the medicinal plant *U. indica* was chosen for the isolation of the endophytic fungus *C. globosum* from its roots. *C. globosum* is known as a biocontrol fungus because it can kill pathogens due to the production of secondary metabolites having strong antifungal and insecticidal potential [5]. This work is the first report of the isolation of endophytic fungi *C. globosum* from *U. Indica*, a potent medicinal plant, and checking the antifungal activity. The purpose of the study is to isolate the endophytic fungus *Chaetomium globosum* pure culture from the medicinal plant *U. indica*, and the preparation of extract was done. Testing of extract should be done to check the presence of essential compounds present in the fungus responsible for antimicrobial and antifungal activities. The best extract among the three extracts should be taken further for fungicide studies. The present study shows the isolation and characterization of *C. globosum* and the analysis of different compounds present in methanolic, hexane, and chloroform extracts through GC-MS.

*Chaetomium globosum* (*C. globosum*) is an endophytic fungus commonly present in the roots of medicinal plants. This fungus produces different secondary metabolites with high antifungal potential, especially against phytopathogenic fungi [6–8].

## 2. Materials and Methods

### 2.1. Isolation and Identification of the Endophytic Fungus

The endophytic fungus (*C. globosum*) isolated from the roots of *U. indica* was collected from the botanical garden of Shoolini University, (Himachal Pradesh), India. *U. indica* belongs to the family of *Liliaceae U. indica* was identified by Professor Sunil Puri Shoolini University, Solan, Himachal Pradesh, India. Roots of *U. indica* were collected in December 2021. Now, the voucher specimen is deposited in BSI Solan. Morphological and molecular identifications of the isolate (MCC 9353) were carried out further for phylogenetic analysis. The isolation of the fungus from the roots was done on a PDA medium (Potato Dextrose Agar).

### 2.2. Morphological Identification

The morphology of the isolated fungus was studied under the microscope through the scotch tape method [9]. The morphological identification was authenticated by the plant tissue culture Lab, Department of biology and biotechnology, Shoolini University, District Solan, Himachal Pradesh. The cut tape is about 2-inches long and touches the tape to a fungal colony in a culture dish opened in a biological safety cabinet. Use a second applicator stick to press the tape down onto the mycelial surface, lift the tape flag with attached fungal elements from the colony and lower it onto a glass slide, add drops of cotton blue dye, and then cover it with a coverslip. The completed mount is now ready for microscopic study.

### 2.3. Molecular Identification

For molecular identification of the fungus, DNA extraction was done. For genomic DNA extraction, around 5 g of fungal mycelia were used. The extraction was done according to [10]. Internal transcribed spacer (ITS) primers were used for the amplification of nuclear ribosomal DNA. The nuclear ribosomal DNA ITS of the fungal isolates were amplified using the forward primer, ITS_1-_ forward primer (5′-AAACCATTGGTGAACGTTA-3′) and ITS_4_ reverse primer (3′ACCGAGGTCACCTTG-5′) [11]. The final reaction volume was 25 *μ*L, containing 2 *μ*g of genomic DNA, Primers 0.1-0.5 *μ*L of 2× PCRBioTaq Mix, 0.4 *μ*L. PCR was done by using a Thermal cycler (Applied Biosystems, India), PCR cycle occurs at 35 cycles programmed for denaturation of 5 min at 94°C, denaturation of 45 s at 94°C, annealing of 45 s at 55°C, and extension of 120 sec at 72°C. PCR products were separated using a 1% agarose gel in 1× TAE buffer (90 mM Tris-acetate and 2 mM EDTA, pH 8.0), stained with ethidium bromide (1 *μ*g/mL) and recognized using Gel doc. PCR products were sent to Eurofins Bangalore, India, for sequencing.

### 2.4. Extract Preparation of *C. globosum*

Crude extracts of *C. globosum* were prepared according to [12] to fifty mL of potato dextrose broth (PDB) being inoculated with two discs (8 mM in size) of *C. globosum* grown on Potato Dextrose Agar (PDA) plates. The flasks were then incubated for 5 days at 25°C as stationary cultures. After the growth of fungal, biomass was filtered through Whatman filter paper no. 1. The filtrate obtained was extracted twice with an equal volume of chloroform, hexane, and methanol to obtain three different extracts. The solvent layer was evaporated using a hot air oven at 40°C [13].

### 2.5. Antagonistic Activity against Different Pathogens

*C. globosum* antagonistic effects against different pathogenic fungi including *Fusarium oxysporum*, *Rosellinia necatrix*, *Cladosporium xanthochromaticum*, and *Sclerotinia sclerotiorum* were evaluated using the dual-culture technique [2]. In this method, 2 mM-sized discs of the culture of *C. globosum* (5-day-old culture) and the same-sized discs of pathogenic fungi were placed on opposite sides of 90 mM petri plates containing PDA. As controls, only pathogens are used. The experiment was repeated three times, and the culture was incubated for seven days at 25°C. After 7 days of incubation, the area between the two colonies at the interaction point was measured, the growth of colonies of the tested fungi and antagonist was observed and photographed, antagonistic activity was repeated thrice, and mean standard deviation was observed for the results [2].

### 2.6. Antifungal Activity of Different Extracts of *C. globosum*

The antifungal activity of all the extracts of *C. globosum* was performed using the poison food technique [14, 15]. Briefly, each extract at 20 mg/mL of extract dissolved by dimethylsulfoxide (DMSO), 10% was added to sterilize the PDA medium. Pathogenic fungal discs of 6 mM in diameter were placed in the centers of petri dishes. Plates without extracts and 5 mg/mL of hygromycin were used as negative and positive controls, respectively. After placing the fungal disc, the plates were incubated at 28 ± 2°C for 7-8 days. Then, the circular growth of mycelium was calculated. The growth results were compared with the negative control. The experiment was repeated three times, and the mean of the readings was taken for further calculations. The percent inhibition was calculated using the following formula:

(1) $$\begin{array}{c} L=\frac{\left[C-T\right]}{\left[C\right]}\times 100, \end{array}$$

where *L*, *C*, and *T* are the percent inhibition, colony radius in the control plate, and the radial growth of the pathogen in the presence of *C. globosum* extracts, respectively [15].

### 2.7. Antioxidant Activity of Different Extracts of *C. globosum*

2,2-diphenyl-1-picrylhydrazyl (DPPH) radical scavenging activity assay was done to check the antioxidant capacity of methanol, chloroform, and hexane extracts of the fungus according to Joshi et al. [16], with some modifications. Briefly, a stock solution of DPPH (0.1 mM) was prepared in methanol and kept in the dark at room temperature for 2 hours. Different concentrations of extracts (20-160 *μ*g/mL) were mixed with 150 *μ*L of DPPH for checking the radical scavenging activity of them and kept in dark for 30 min at room temperature. Absorbance was measured at 517 nm using Systronics UV-vis double beam spectrophotometer 2205. DPPH free radical scavenging activity was expressed as the percentage inhibition of each extract and by calculating the IC_50_ value. The antioxidant activity was calculated using the following formula equation:

(2) $$\begin{array}{c} \%\text{Scavenging activity}=\frac{\left[\left(\text{Ao}-A1\right)\right]}{\left[\text{Ao}\right]}\times 100, \end{array}$$

where Ao and *A*1 are the absorbance of the blank and test/positive control, respectively.

### 2.8. GC–MS Analysis

Crude extracts (i.e., hexane, methanol, chloroform) of the *C. globosum* fungus were further analyzed for GC-MS, which is used for compound detection. GC-MS was carried out by using a Thermo Trace 1300GC coupled with a Thermo TSQ 800 Triple Quadrupole MS with a column (30^∗^0.25 mM,0.25 *μ*M). The samples were injected in split mode as 10 : 1. Different steps are involved in the reaction to carry out different compounds present in the extracts; the initial temperature is 60°C for 3 minutes, the oven temperature is 280°C at an increased rate of 15°C for 19 minutes, injection port temperature is 260°C for 1 minute, helium used for 1 mL, the flow rate is 1 min, and ionization voltage is 70 eV. MS scans at speeds ranging from 50 to 650 m/z. The identification of each compound was based on the comparison of the mass spectra (MS) spectra computer matching with standard reference databases NIST Ver.2.1 MS.

### 2.9. Statistical Analysis

All the experiments were observed in triplicate, and the obtained data were analyzed statistically with the help of GraphPad Prism 5.02 software, and the results are presented as mean ± standard deviation.

## 3. Result and Discussion

### 3.1. Isolation and Characterization of Endophytic Fungus

**Figure 1:**
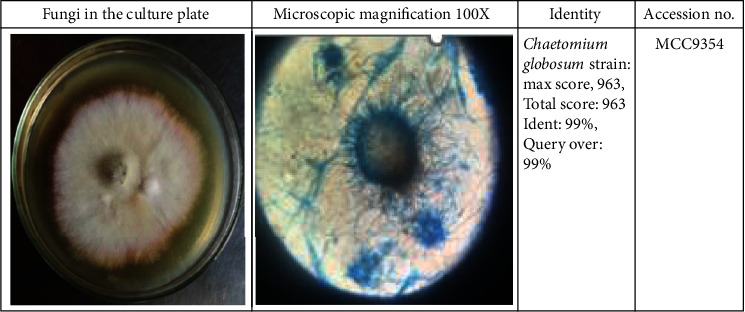
*Chaetomium globosum* isolated from *Urginea indica* and its morphological identification under a microscope (Molecular confirmation of *C. globosum* using the universal primer pair ITS1 and ITS4 which had an amplicon of 560 bp).

After isolation, identification of the fungus strain was done. Molecular identification was done by rDNA sequencing of the ITS region, and morphological identification was done through the scotch tape method. The sample was identified as one of the *Chaetomium* species previously mentioned in the literature.  Figure 1 is the picture of the microorganism observed and photographed under 100×. This identification shows the 99% similarity of the isolate with the fungus identified. Then, the fungus was deposited in NCIM Pune to get an accession number (MCC9354). The phylogenetic relationship of the isolate with its related fungus is shown in Figure 1. The isolated endophytic fungus belongs to the phylum *Ascomycota* [17]. *C. globosum* was isolated from different parts of the medicinal plants earlier, such as *Moringa oleifera* [18], *Amaranthus viridis* [19], and barnyard grass [7]. [20] isolated 10 isolates of *C. globosum* from different medicinal plants and identified these isolates by morphological and molecular methods. For the first time, we isolated the endophytic fungus *C. globosum* from the roots of *U. indicia.* This is the first report of the isolation of *C. globosum* from this plant.

### 3.2. Molecular Identification of the Fungal Sample Based on Its Region-Specific Primers

**Figure 2:**
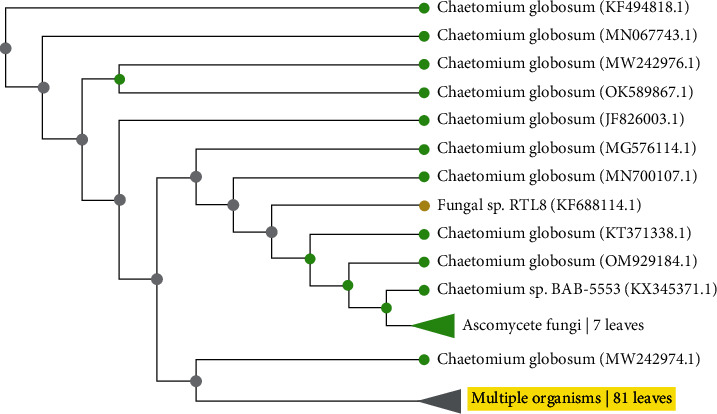
Phylogenetic tree showing the relationship of *Chaetomium globosum* with other related fungal species with reference sequences retrieved from NCBI (National Center for Biotechnology Information).

**Figure 3:**
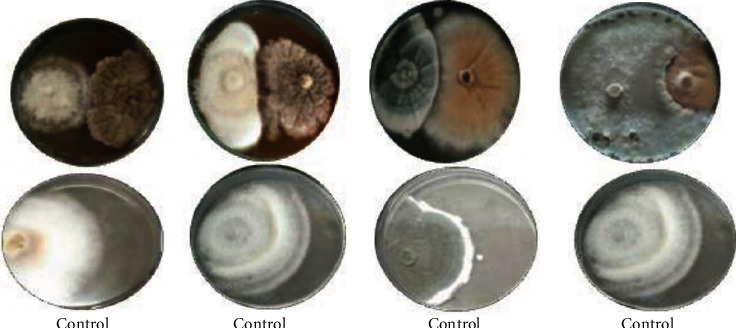
Antagonistic effects of *Chaetomium globosum* against pathogenic fungi (a) *Rosellinia necatrix*, (b) *Fusarium oxysporum*, (c) *Cladosporium xanthochromaticum*, and (d) *Sclerotinia sclerotiorum.*

The growth of the fungus was observed after 4-5 days of inoculation. For identification at the molecular level, the total genomic DNA was isolated as per the protocol given by [21]. A PCR was performed. The PCR cycle occurs every 35 cycles and the following steps are involved in the completion of this cycle: denaturation at 94°C for 5 min, again denaturation at 94°C for 45 sec, annealing at 55°C for 45 sec, extension at 72°C for 120 sec, and again extension at 72°C for 10 min. For PCR genomic DNA 1-2 *μ*g, primers forward and reverse 0.1-0.5 *μ*M, TE buffer100 *μ*M, Mg^2+^,1.5 mM, and dNTP 100 mM can be seen in Figure 2. DNA bands (PCR products) in the gel were shown. The results revealed that molecular confirmation of *C. globosum* using the universal primers ITS1 and ITS4, which had an amplicon of 500-560 bp, [22] also reported that with the combination of universal primers, ITS1 and ITS4, sequences amplicon sizes of 500-560 bp were observed. After BLAST analysis, the sample showed 100% similarity with *Chaetomium* spp. strain CBS 105.40 with accession number MH856051.1. Further phylogenetic analysis was done by MEGA 7.0.9.  Figure 3 shows the similarity percentage of *C. globosum*, and Figure 2 shows the phylogenetic relationship of the fungus.

### 3.3. Antagonistic Effects of *C. globosum*

Antagonistic activity of *C. globosum* against different phytopathogenic fungi through a bicultural method such as *Fusarium oxysporum*, *Sclerotinia sclerotiorum*, *Rosellinia necatrix*, and *Cladosporium xanthochromaticum* has been shown in Figure 3. In the control plate, pathogenic fungi grow faster and remarkably form larger colony diameters with a mean of 9.97 cm; while in bicultural plates, these pathogenic fungi form small colonies with a mean diameter of 4 cm for *Fusarium oxysporum*, 3.8 cm for *Cladosporium xanthochromaticum*, 3.2 cm for *Sclerotinia sclerotiorum*, and 3.9 cm for *Rosellinia necatrix*. The highest inhibition of mycelial growth of pathogens against *Chaetomium globosum* was shown by *Fusarium oxysporum* as (80.4%), followed by *Rosellinia necatrix* (78.32%), *Cladosporium xanthochromaticum* (68.69%), and *Sclerotinia sclerotiorum* (60.66%). The antagonism effect of *Chaetomium globosum* was reported earlier by [2] showed inhibition against the pathogens in the bicultural method *F. graminearum*, 50.2% and *S. sclerotiorum*, 78.9%. Strongest inhibition has been displayed against *S. sclerotiorum*, i.e., 78.9% which is high as compare to our results where the highest inhibition of 80.4% was shown against *Fusarium oxysporum* as compare to *Sclerotinia sclerotiorum* which is 60.66%. Antagonistic activity of *Chaetomium globosum* through biculture test against *Fusarium oxysporum* (67.25%) has been previously reported by Phong et al. [23], and our study displays the antagonistic activity of 80.4% against *Fusarium oxysporiu*, i.e., which is higher as compared to their study.

### 3.4. Antifungal Activity of *C. globosum*

**Figure 4:**
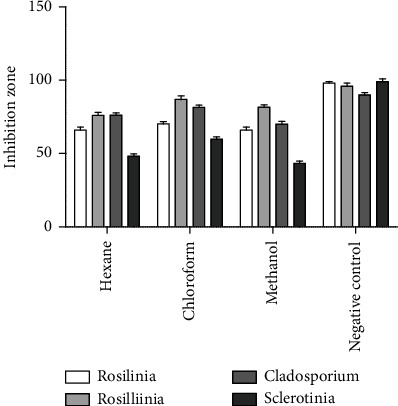
Inhibition has been shown by *Chaetomium globosum* extracts (hexane, methanol, and chloroform) against different pathogens (*Fusarium oxysporum*, *Rosellinia necatrix*, *Cladosporium xanthochromaticum*, and *Sclerotinia sclerotiorum*).

Metabolites produced by *C. globosum* in culture inhibited the growth of test pathogens. All the crude extracts of the fungus showed significant inhibition against the test pathogens *F. Oxysporum*, *R. necatrix*, *C. xanthochromaticum*, and *Sclerotinia sclerotiorum*. *Rosellinia necatrix* is a cosmopolitan fungus that is found all over the world and has a high ability to kill infected trees Attri and Kulshrestha [24]. *Fusarium* can infect a variety of crops, including rice, ornamentals, wheat, and all horticultural crops [25]. The results of this assay are shown in Table 1. All the extracts at 500 *μ*g/mL showed moderate to high antifungal activity against all the pathogens. Methanolic extract showed maximum inhibition against all pathogens as compared to the chloroform and hexane extracts [26]. Extracts were found to be effective against all tested fungal isolates. Antifungal activity of ethanol, methanol, and butanol extracts of *C. globosum* was studied previously by [27]; results of the study stated that hexane extract shows less inhibition as compared to ethyl acetate and ethanol extracts, showing 80% inhibition against *S*. *sclerotiorum.* The isolated fungus shows outstanding antifungal activity against different pathogens. *Chaetomium globosum* on PDA plates shows potent antagonistic activity against different pathogens that result in the exposition of positive biological activities [28]. Hexane extract of *Chaetomium globosum* has been reported as an antifungal against *Sclerotiorum* and *Botrytis cinerea* [29]. Strong antifungal activity of *C. globosum* against *Fusarium oxysporum* which causes fusarium wilt of tomato [30]. Inhibition of *Sclerotinia sclerotiorum* by solvent extract of *C. globosum*, i.e., hexane, methanol, and ethyl acetate extract were shown by Kumar et al. [31].Though we find polar extract, i.e., methanol and chloroform were more active than hexane against *Fusarium oxysporum*, *Rosellinianecatrix*, *Cladosporium xanthochromaticum*, and *Sclerotinia sclerotiorum.* Our study also shows less inhibition in hexane extract as compared to methanol, and chloroform extracts show 80-90% inhibition. The graphical representation of shows inhibition of all the extracts is shown in Figure 4.

### 3.5. Antioxidant Activity

**Figure 5:**
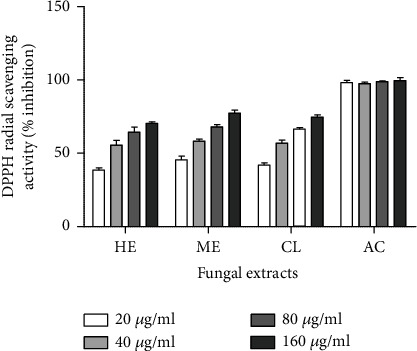
Free radical scavenging activity of different fungal extracts (HE represents hexane extract; ME represents methanol extract; CL represents chloroform extract) and the standard drug (AC represents ascorbic acid).

In the present study, all the crude extracts of *C. globosum* showed significant DPPH radical scavenging activity. The antioxidant potential of different extracts was examined at different concentrations (20, 40, 80, and 160 *μ*g/mL). Methanol extracts showed remarkable antioxidant activity ranging from 55 to 70% inhibition, while the hexane and chloroform extracts showed a narrow spectrum of radical scavenging activity ranging from 30 to 50% inhibition (Figure 5). The standard drug, ascorbic acid, showed 97% inhibition radical scavenging activity. Antioxidant capacity was evaluated as IC_50_ values were also examined, the lower the IC_50_ value, the higher the scavenging activity, IC_50_ of methanolic extract was 37.61 ± 1.37, and the chloroform extract was 40.82.±3.60, hexane extract was 45.20 ± 2.54, and IC_50_ of ascorbic acid was 50 ± 1.2. DPPH scavenging activity of chloroform extract was checked by Kaur et al. [18]. At 20-100 *μ*g/mL concentration, percentage inhibition at lower concentration, i.e., 20 *μ*g/mL was 30.59% inhibition, our chloroform extract shows 34.5% inhibition at 20 *μ*g/mL, which expressively increased by the increase in the concentration of chloroform extract. The IC_50_ value was 45 *μ*g/mL, whereas our result shows a 40.82 *μ*g/mL IC_50_ value which is almost closer to their results. Antioxidant DPPH scavenging activity of polysaccharide produced by *C. globosum* was checked by [1] at 0.5, 1.0, 2.0, 3.0, 4.0, and 5.0 mg/mL, activity showed 83.08% inhibition. In 2014, Awad et al. [32] studied the antioxidant activity of petroleum ether, ethyl acetate, diethyl ether, chloroform, and ethyl acetate extracts; all extracts shows 53, 81.9, and 93.9% inhibition at 10 mg, 50 mg, and 100 mg concentration. Results stated that as compared to other extracts, petroleum ether and ethyl acetate extract showed the highest antioxidant activity. There are lots of metabolites present in the extract of fungi; and because of the presence of these metabolites, extract shows the strongest antioxidant activity. In the current study, the polar extract, i.e., methanol and chloroform showed 55-70% inhibition and 30-50% inhibition scavenging potential, whereas the nonpolar extract, i.e., hexane showed 40% scavenging potential for DPPH.

### 3.6. GC-MS Analysis

**Figure 6:**
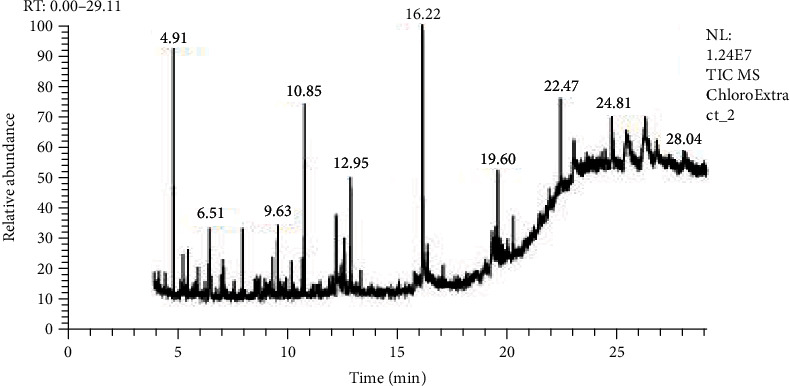
GC-MS analysis of *C. globosum* chloroform extract.

**Figure 7:**
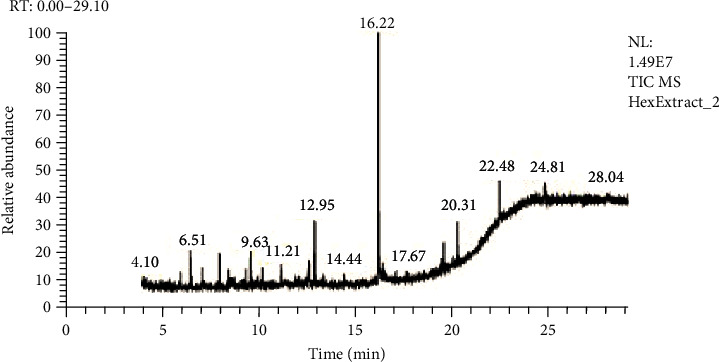
GC-MS analysis of the hexane extract of *Chaetomium globosum.*

**Figure 8:**
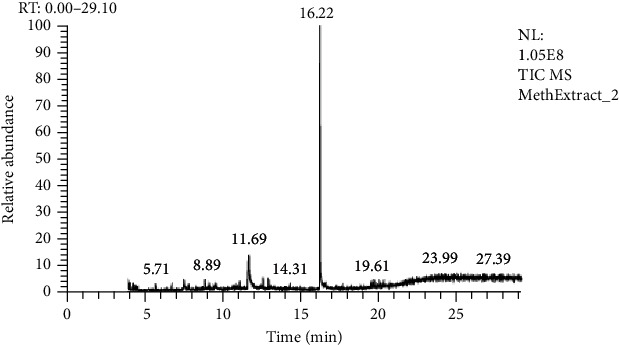
GC-MS analysis of the methanol extract of *Chaetomium globosum.*

GC-MS analysis was done by Hateet [33] with few modifications. The presence of bioactive compounds in all three extracts of *C. globosum* was identified by GC-MS, which showed the existence of numerous compounds with consistent peaks at different retention times as shown in Figures 6–8. Compounds detected through GC-MS were shown in Tables 2–4 for chloroform, hexane, and methanol extracts, respectively. Bioactive compounds analysis of *C. globosum* chloroform extract through GC-MS previously reported by Kaur et al. [18], major compounds detected in chloroform extract were phenol, 2,4bis(1,1dimethylethyl), E-14-hexadecenal, 10-heneicosene (c, t), 3-eicosene, and 1-heneicosanol. In our study, the major compounds detected in chloroform extract were acetic acid, diethoxy-, ethyl ester, 2,2-bis(ethylsulfonyl)propane, 3-methyl-2-(2-oxopropyl) furan, 2,2-dimethyl-propyl 2,2-dimethyl-propanesulfinyl sulfone, decane, 2,4,6-trimethyl, dodecane, 2,6,10-trimethyl, and 14-heptadecenal. Kanjana et al. [34] isolate the compounds through GC-MS; the major compounds found in the ethylene extract were 5-isopropyl-2-methylbicyclo[3.1.0]hex-2ene; propane, 1,1,3-triethoxy-; 2,6-Octadienal, 3,7-dimethyl-, (Z)-; 2-propenal, 3-phenyl-; thymol; 2-cyclohexen-1-one, 2-methyl-5(1-methylethenyl)-; 5-allyl-2-methoxyphenol; dodecanoic acid; n-hexadecanoic acid; 9-octadecenoic acid, (E)-; and decanedioic acid, bis(2-ethylhexyl) ester. In another study, GC-MS analysis of ethyle acetate extract of *Chaetomium globosum* was performed by Kamat et al. [6], the major compounds isolated were 2,2-diethylacetamide, hexadecanoic acid, 9,12-octadecadienoic acid (Z,Z), trans-9-octadecenoic acid, octadecanoic acid, chrysin, and a propyl ester of octadecanoic acid. In our study, we use three different extracts of *C. globosum*, and the major chemical constituents present in the methanol extract (1,3-oxathiolane, 1,3-cyclopentadiene, 5-(1-methylethylidene), 5,9-hexadecadienoic acid, methyl ester, decane, 1-methylene-2-benzyloxy-cyclopr opane, 2,2-diethyl-N-ethylpyrrolidine, 1,2,3-thiadiazole, 5-methyl, chloroform extract (acetic acid, diethoxy-, ethyl ester, 2,2-bis(ethylsulfonyl)propane, 3-methyl-2-(2-oxopropyl) furan, 2,2-dimethyl-propyl 2,2-dimethyl-propanesulfinyl sulfone, decane, 2,4,6-trimethyl, dodecane, 2,6,10-trimethyl, and hexane extract(3-hexanone, 4,4-dimethyl, decane,2,6-dimethyldecane, decane, 2,4,6-trimethyl, decane, 2,4,6-trimethyl, 1-butanesulfinamide, 1,1,2,2,3,3,4,4,4-nonafluoro-N-methyl, decane, 2,4,6-trimethyl, ether, hexyl pentyl, heptadecane, 1,8-naphthyridine, 2,4-dimethyl).

This conforms with the previous investigation done by [35] on numerous endophytic fungi. Bioactive compounds present in the methanolic, chloroform, and hexane extracts have been analyzed by using GC-MS which displayed the existence of several compounds that might be responsible for bioactivities. The total compound was identified at 56.2% in chloroform extract, 54.72% in hexane extract, and 65% in methanol extract.

## 4. Conclusion

The present work is the first comprehensive study of the antioxidant and antifungal activities of *Chaetomium globosum* isolated from *Urginea Indica.* Different extracts (methanol, hexane, and chloroform) were prepared from the *C. globosum* fungus and checked for antifungal activities. Methanol and chloroform extracts showed good antifungal activity as compared to hexane extract. The antioxidant activity of all three extracts was measured by the DPPH (2,2-diphenyl-1-picrylhydrazyl) assay, methanol and chloroform extract showed the highest scavenging potential hexane extract showed the lowest scavenging potential for DPPH.

In addition, GC-MS analysis was also done to check the presence of various bioactive compounds in different extracts of *Chaetomium globosum*. This study concluded that the presence of bioactive compounds in fungi helps in the inhibition of pathogens causing disease in plants. Further studies are required to study the biocontrol activity of *C. globosum* against several plant diseases and to elucidate its mechanism of action in disease control programs. Endophytic *C. globosum* possesses good bioactive potential and can be used further for the development of bioactive drugs for pharmaceutical and medical applications.

## Figures and Tables

**Table 1:** Percentage inhibition of hexane, methanol, and chloroform extract of *Chaetomium globosum* against different pathogens (*Fusarium oxysporum*, *Rosellinia necatrix*, *Cladosporium xanthochromaticum*, and *Sclerotinia sclerotiorum*).

Extracts	Pathogens	% inhibition mean ± SD	Inhibition zone (mM)
Methanolic extract	*Fusarium oxysporum*	86 ± 1.52	10.4
	*Cladosporium xanthochromaticum*	76 ± 2	9.2
	*Rosellinia necatrix*	81 ± 1.52	9.8
	*Sclerotinia sclerotiorum*	46 ± 1.52	5.6

Chloroform extract	*Fusarium oxysporum*	81 ± 2.5	9.8
	*Cladosporium xanthochromaticum*	70 ± 1.52	8.4
	*Rosellinia necatrix*	76 ± 2	9.2
	*Sclerotinia sclerotiorum*	59 ± 2	7.1

Hexane extract	*Fusarium oxysporum*	70 ± 1.5	8.4
	*Cladosporium xanthochromaticum*	66 ± 2	8
	*Sclerotinia sclerotiorum*	48 ± 1.5	5.8
	*Rosellinia necatrix*	66 ± 2	8

Negative control	*Fusarium oxysporum*	91.26 ± 2	11
	*Rosellinia necatrix*	90 ± 1	10.9
	*Cladosporium xanthochromaticum*	88.92 ± 1.5	10.6
	*Sclerotinia sclerotiorum*	91.67 ± 1.2	11.2

**Table 2:** The presence of different bioactive compounds in the chloroform extract of *C. globosum* detected through GC-MS.

Name of compound	Molecular formula	MW	RT	Peak %	Nature of compound
Acetic acid, diethoxy-, ethyl ester	C_8_H_16_O_4_	176	4.91	6.09	Ester

2,2-Bis(ethylsulfonyl)propane	C_7_H_16_O_4_S_2_	228	5.33	1.31	Alkane

3-Methyl-2-(2-oxopropyl)furan	C_8_H_10_O_2_	138	5.54	1.44	Aldehyde

2,2-dimethyl-propyl 2,2-dimethyl-propanesulfinyl sulfone	C_10_H_22_O_3_S_2_	254	6.02	1.34	Sulfone

Decani, 2,4,6-trimethyl-	C_13_H_28_	184	6.51	3.29	Alkane

Dodecane, 2,6,10-trimethyl-	C_15_H_32_	212	7.12	1.06	Alkane

14-Heptadecenal	C_17_H_32_O	252	7.17	1.08	Steroid

4,5′-Dibenzamido-1,1′-iminodianth quinone	C_42_H_25_N_3_O_6_	667	8.03	2.11	Quinone

1-Iodo-2-methylnonane	C_10_H_21_I	268	10.2	1.32	Alkane

1,3,4,6-Hexanetetrone, 1-(4-methoxyphenyl)-6-phenyl-1,4-dioxane,	C_19_H_16_O_5_	324	10.7	1.38	Heterocyclic

1,1,1,3,5,5,5-Heptamethyltrisiloxane	C_7_H_22_O_2_Si_3_	222	28.12	1.23	Alkane

1,2,4-Benzenetricarboxylic acid, 1,2-dimethyl ester	C_11_H_10_O_6_	238	21.96	1.29	Ester

1-Pentanol, 2,3-dimethyl-	C_7_H_16_O	116	20.04	1.92	Alcohol

1,3-Dibromo-1,3-dichloropropane	C_3_H_2_Br_2_C_l2_O	282	10.85	5.48	Alkane

2-Hexyl-1-octanol	C_14_H_30_O	214	12.09	1.34	Alcohol

1-Dodecanol	C_12_H_26_O	186	12.28	3.72	Alcohol

1-Octadecanesulphonyl chloride	C_18_H_37_ClO_2_S	352	12.50	1.02	Alkyl

2,4-Di-tert-butylphenol	C_14_H_22_O	206	12.95	4.54	Phenol

1,3-Methanopentalene, octahydro-	C_9_H_14_	122	16.22	12.05	Alkene

6-Tetradecanesulfonic acid, butyl ester	C_18_H_38_O_3_S	334	16.44	1.71	Ester

**Table 3:** Presence of different bioactive compounds in hexane extract of *Chaetomium globosum* detected through GC-MS.

Name of compound	Molecular formula	MW	RT	Peak %	Nature of compound
3-Hexanone, 4,4-dimethyl-	C_8_H_16_O	128	6.02	1.50	Ketone

Decane	C_10_H_22_	142	6.51	3.58	Alkane

2,6-Dimethyldecane	C_12_H_26_	170	6.59	1.04	Alkane

Decane, 2,4,6-trimethyl-	C_13_H_28_	184	7.12	1.33	Alkane

1,2-Bis(3,5-dimethylphenyl)-diazene 1-oxide	C_16_H_18_N_2_O	254	8.02	2.75	Alkene

Decane, 2,4,6-trimethyl-	C_13_H_28_	184	8.49	1.53	Alkane

1-Butanesulfinamide, 1,1,2,2,3,3,4,4,4-nonafluoro-N-methyl-	C_5_H_4_F_9_NOS	297	9.27	0.99	Amines

1,1,1,3,5,5,5-Heptamethyltrisiloxane	C_7_H_22_O_2_Si_3_	222	28.04	1.23	Alkane

Ether, hexyl pentyl	C_11_H_24_O	172	9.63	2.68	Ether

Heptadecane	C_17_H_36_	240	11.21	1.99	Alkane

1-Pentanol, 2,3-dimethyl-	C_7_H_16_O	116	11.38	1.13	Alkyl

1,8-Naphthyridine, 2,4-dimethyl-	C_10_H_10_N_2_	158	12.13	1.99	Alkyl

3-Methyl-2-(2-oxopropyl)furan	C_8_H_10_O_2_	138	12.50	1.23	Heterocyclic

2,4-Di-tert-butylphenol	C_14_H_22_O	206	12.95	6.57	Phenol

1,3-Cyclopentadiene, 5-(trans-2-ethyl-3-methylcyclopropylidene)-	C_11_H_14_	146	13.07	1.02	Alkene

6-Tetradecanesulfonic acid, butyl ester	C_18_H_38_O_3_S	334	13.39	1.27	Ester

3,6-Octadienal, 3,7-dimethyl-	C_10_H_16_O	152	16.22	27.82	Alkyl

1,2,4-Oxadiazolidin-5-one,2-tert-butyl-3-(tertbutylimino)-4-phenyl-	C_16_H_23_N_3_O_2_	289	19.61	2.56	Alkyl

2,6,10,14-Tetramethylpentadecan-2 -ol	C_19_H_40_O	284	20.04	1.75	Alcohol

1-Hexyl-1-nitrocyclohexane	C_12_H_23_NO_2_	213	20.31	3.66	Alkane

**Table 4:** Presence of different bioactive compounds in methanol extract of *Chaetomium globosum* detected through GC-MS.

Name of compound	Molecular Formula	MW	RT	Peak %	Nature of compound
1,3-Oxathiolane, 2-[[(2-chloroethyl) thio] methyl]-2- methyl-	C_7_H_13_C_l_OS_2_	212	4.09	0.79	Alkane

1,3-Cyclopentadiene, 5-(1-methylethylidene)-	C_8_H_10_	106	4.31	0.98	Alkene

1,3-Oxathiolane	C_3_H_6_OS	90	4.44	1.33	Alkane

5,9-Hexadecadienoic acid, methyl ester	C_27_H_50_O_2_	406	5.00	0.44	Carboxylic

Decane	C_10_H_22_	142	5.71	0.58	Alkane

1-Methylene-2-benzyloxy-cyclopropane	C_11_H_12_O	160	6.45	0.44	Alkane

2,2-Diethyl-N-ethylpyrrolidine	C_10_H_21_N	155	6.74	0.70	Amines

1,2,3-Thiadiazole, 5-methyl	C_3_H_4_N_2_S	100	3.30	7.53	Azole

2-Butanol, 2-nitroso-, acetate (ester)	C_6_H_11_NO_3_	145	7.83	1.08	Alcohol

1-Undecanol	C_11_H_24_O	172	8.37	0.37	Fattyalcohol

1,3-Diazacyclooctane-2-thione	C_6_H_12_N2S	144	9.55	2.27	Ketone

1-Allyl-cyclohexane-1,2-diol	C_9_H_16_O_2_	156	10.68	1.36	Alcohol

1-Butanesulfinamide, 1,1,2,2,3,3,4,4,4-nonafluoro-N-me thyl-	C_5_H_4_F_9_NOS	297	10.96	0.54	Alkyl

1-Dodecanol, 3,7,11-trimethyl-	C_15_H_32_O	228	11.11	1.13	Alkyl

Benzo[3,4]cyclobuta[1,2-b]-1,4-dio xin, 2,3,4a,4b,8a,8b-hexahydro-	C_10_H_12_O_2_	164	11.69	17.16	Aromatic aldehydes

1-Propanol, 2,2-dimethyl-	C_5_H_12_O	88	12.46	0.94	Neopentyl alcohol

1,3-Cyclopentanedione, 2-ethyl-4-propyl-	C_10_H_16_O_2_	168	12.61	2.02	Ketone

2,4-Di-tert-butylphenol	C_14_H_22_O	206	12.95	1.39	Phenol

1-Allyl-cyclohexane-1,2-diol	C_9_H_16_O_2_	156	13.07	0.84	Alcohol

1-Eicosanol	C_20_H_42_O	298	14.31	0.90	Long-chain fatty alcohols

3,6-Octadienal, 3,7-dimethyl-	C_10_H_16_O	152	47.85	16.22	Acyclic monoterpenoid

1,3-Cyclopentadiene, 1,2-dimethyl-	C_7_H_10_	94	16.58	0.95	Cyclic diene

2-Acetyl-1-pyrroline	C_6_H_9_NO	111	17.28	0.75	Ketone

1-Nonadecene	C_19_H_38_	266	17.67	0.40	Alkene

1,9Diazaspiro(4,4)nonane-2,8-dione	C_7_H_10_N_2_O_2_	154	18.22	0.48	Ketone

1,1,1,3,5,5,5-Heptamethyltrisiloxane	C_7_H_22_O_2_Si_3_	222	18.85	0.57	Alkane

(2*R*,3*S*,4*S*)-2-(hydroxymethyl)-3,4-dihydro-2*H*-pyran-3,4,5-triol	C_17_H_24_O_3_	276	19.49	0.71	Alcohol

1,4-Benzenediol, 2-(5,16-dihydroxy-3,7,11,15-tetra methyl-2,6,10,14-hexadecatetraeny l)-6-methyl-, (E,Z,E,E)-	C_27_H_40_O_4_	428	19.61	0.98	Alcohol

1-Aminononadecane, N-trifluoroacetyl-	C_21_H_40_F_3_O	379	19.90	0.48	Alkane

2-Methyl-5-oxo-7-(2-phenyl-1,3-dioxolan-2-yl) heptanenitrile	C_17_H_21_NO_3_	287	20.07	0.67	Alkyl

## Data Availability

The data used to support the findings of this study are available from the corresponding author upon request.
